# Dynamic Causal Modeling of the Relationship between Cognition and Theta–alpha Oscillations in Adults with Down Syndrome

**DOI:** 10.1093/cercor/bhz043

**Published:** 2019-03-16

**Authors:** Sarah Hamburg, Richard Rosch, Carla Marie Startin, Karl John Friston, André Strydom

**Affiliations:** 1 Division of Psychiatry, Faculty of Brain Sciences, University College London, 149 Tottenham Court Road, London, UK; 2 Department of Forensic and Neurodevelopmental Sciences, Institute of Psychiatry, Psychology and Neuroscience, Kings College London, London, UK; 3 The London Down Syndrome Consortium (LonDownS), London, UK; 4 MRC Centre for Neurodevelopmental Disorders, Institute of Psychiatry, Psychology and Neuroscience, King's College London, London, UK; 5 Wellcome Centre for Human Neuroimaging, Institute of Neurology, University College London, 12 Queen Square, London, UK

**Keywords:** alpha, cognition, Down syndrome, dynamic causal modeling, intellectual disability

## Abstract

Individuals with Down syndrome (DS) show high inter-subject variability in cognitive ability and have an ultra-high risk of developing dementia (90% lifetime prevalence). Elucidating factors underlying variability in cognitive function can inform us about intellectual disability (ID) and may improve our understanding of factors associated with later cognitive decline. Increased neuronal inhibition has been posited to contribute to ID in DS. Combining electroencephalography (EEG) with dynamic causal modeling (DCM) provides a non-invasive method for investigating excitatory/inhibitory mechanisms. Resting-state EEG recordings were obtained from 36 adults with DS with no evidence of cognitive decline. Theta–alpha activity (4–13 Hz) was characterized in relation to general cognitive ability (raw Kaufmann’s Brief Intelligence Test second Edition (KBIT-2) score). Higher KBIT-2 was associated with higher frontal alpha peak amplitude and higher theta–alpha band power across distributed regions. Modeling this association with DCM revealed intrinsic self-inhibition was the key network parameter underlying observed differences in 4–13 Hz power in relation to KBIT-2 and age. In particular, intrinsic self-inhibition in right V1 was negatively correlated with KBIT-2. Results suggest intrinsic self-inhibition within the alpha network is associated with individual differences in cognitive ability in adults with DS, and may provide a potential therapeutic target for cognitive enhancement.

## Introduction

Down syndrome (DS) is caused by an extra copy of chromosome 21 and is the most common genetic cause of intellectual disability (ID) worldwide, affecting 1 in 800 births (de Graaf et al. 2015). While almost all individuals with DS have an ID (IQ < 70), there is a high degree of variation in cognitive ability between individuals (Startin et al. 2016). Mechanisms underlying differences in cognitive ability in this population are as yet undetermined.

Additionally, adults with DS are at an ultra-high risk of Alzheimer’s disease (AD) (lifetime prevalence 90% (McCarron et al. 2014; Zis and Strydom 2018)) attributed to triplication of the *APP* gene which is located on chromosome 21. A more in-depth understanding of how variability in brain function in this group of individuals, who are genetically at risk of AD, is associated with cognitive ability may provide mechanistic insights linking genetic dementia risk and cognitive impairment.

Electroencephalography (EEG) measurements reveal oscillatory brain activity across distinct frequency bands. These bands are believed to represent different dynamic network states and have been associated with a variety of different functions. Alpha oscillations (8–13 Hz) are one of the most prominent and reliably measured signals (Gasser et al. 1985; Vázquez-Marrufo et al. 2017). Alpha oscillations have been shown to modulate perception and are associated with feedback control of sensory information (Jensen et al. 2014; Popov et al. 2017). Consequently, alpha activity may have utility as a marker of intact distributed network activity, and may potentially be associated with cognitive ability.

Consistent with this notion, previous research has suggested adults with DS show atypical alpha-band features compared to adults of the typically-developing (TD) population, including a slower alpha peak frequency (APF), with the APF within the theta range for some individuals (Gunnarson 1945; Ono et al. 1992; Soininen et al. 1993; Murata et al. 1994; Locatelli et al. 1996; Velikova et al. 2011). Furthermore, within adults with DS, individual differences in alpha power and alpha frequency have somewhat inconsistently been associated with cognitive ability (Soininen et al. 1993; Locatelli et al. 1996; Politoff et al. 1996; Medaglini et al. 1997; Velikova et al. 2011), in addition to ageing and cognitive decline (Johanson et al. 1991; Ono et al. 1992; Soininen et al. 1993; Murata et al. 1994; Locatelli et al. 1996; Visser et al. 1996; Medaglini et al. 1997; Katada et al. 2000; Salem et al. 2015). Thus, EEG characteristics in the alpha band may prove useful as biomarkers for whole-brain dysfunction in adults with DS, though further investigation is warranted.

Functionally, alpha band activity may represent modulations of excitatory/inhibitory (E/I) balance of ongoing cortical activity (Peterson and Voytek 2017). Impairments in E/I balance have also been purported as a mechanism contributing to cognitive impairment in DS (Martínez-Cué et al. 2014). Bayesian model inversion schemes, such as dynamic causal modeling (DCM), allow non-invasive inference on parameters of neuronal circuitry from EEG signals (Friston et al. 2003; Kiebel et al. 2009), and have been used to infer E/I parameters from scalp-EEG signals in health and neuropsychiatric conditions (Brown and Friston 2012; Fogelson et al. 2014; Pinotsis et al. 2014; Cooray et al. 2015; Chellappa et al. 2016; Ranlund et al. 2016).

In this study, we quantified whole-scalp resting-state EEG characteristics associated with cognitive ability in a sample of adults with DS without a diagnosis of dementia or noticeable cognitive decline. We used a standard statistical parametric mapping (SPM) approach, a technique for statistically analyzing maps of brain imaging data (Kiebel et al. 2005), to firstly spatially delineate variations in cortical oscillatory activity across theta–alpha frequency bands that were associated with cognitive ability. We then applied DCM for cross-spectral densities (Kiebel et al., 2009; Moran et al., 2009) to infer the cortical circuitry changes underlying these oscillatory correlates; thereby offering insights—at the level of canonical microcircuits (CMCs)—into the neuronal architectures of adults who present with both ID and a genetic susceptibility for dementia.

## Methods

### Ethical Considerations

Ethical approval for the study was obtained from the North Wales West Research Ethics Committee (13/WA/0194). Where individuals had capacity to consent for themselves written informed consent was obtained. Where individuals did not have capacity to consent for themselves, a consultee was asked to sign a form to indicate their decision regarding the individuals’ inclusion based on their knowledge of the individual and his/her wishes, in accordance with the UK Mental Capacity Act 2005.

### Participants

Participants were recruited from an existing pool of the UK adults (aged 16 and over) with DS who had participated in an initial cognitive assessment (see Startin et al., 2016 for further details). All participants taking part in the EEG study detailed here were aged 16 and over and had genetically confirmed trisomy 21 (two participants with non-trisomy 21 DS were excluded). Participants with an acute physical or mental health condition were excluded, as were participants with a clinical diagnosis of dementia or the presence of noticeable cognitive decline associated with dementia, and those who were non-compliant with experimental instructions. The presence of dementia was defined based on informant report of clinical diagnosis. The presence of noticeable cognitive decline was determined using information from the Cambridge Examination of Mental Disorders of Older People with Down Syndrome and Others with Intellectual Disabilities (CAMDEX-DS (Ball et al. 2004)), which is considered a valid and reliable tool for assessing cognitive decline in adults with DS (Ball et al. 2004). All participants were required to show no decline on this questionnaire.

In total, 36 participants aged 16–56 years (*M* = 30.92 years, SD = 11.03; 19 female) meeting the above criteria were selected from the databank.

### Cognitive Assessment

The Kaufmann’s Brief Intelligence Test second Edition (KBIT-2) raw test score was used to provide an estimate of general cognitive ability (Kaufman and Kaufman 2004). The KBIT-2 comprises three subtests, which assess general cognitive abilities through questions relating to verbal knowledge, pattern completion and riddle completion. It provides a raw composite score of verbal and non-verbal abilities, which can then be converted to an age-adjusted IQ score. In the literature, only two tests that measure both verbal and non-verbal abilities have been used in more than one study to assess general cognitive ability in adults with DS—the Wechsler Intelligence Scale for Children Revised (WISC-R; Wechsler 1974) and the KBIT-2 (see review Hamburg et al., submitted). As the WISC-R is designed for use in children, the KBIT-2 was chosen for this study to ensure items were age appropriate. Raw KBIT-2 scores were used, as opposed to age-adjusted IQ scores, due to the high number of participants scoring at floor (i.e., the lowest score possible; IQ of 40) when raw scores were converted to IQ scores. This is a common approach to this issue in DS research (Edgin et al. 2010; Startin et al. 2016).

### EEG Acquisition and Preprocessing Procedure

The initial eyes-closed resting-state EEG paradigm consisted of continuous recording for 5.5 min (i.e., whole-block recording). We use the term resting state to indicate that participants were not asked to complete a specific task (other than closing their eyes). Due to poor participant compliance (i.e., participants had difficulty sitting still and maintaining eye-closure) an amendment was made after 16 participants had been assessed. This involved partitioning the 5.5-min recording into 30 s blocks with a short break (of variable length according to each participant) between blocks, which enabled researchers to reiterate the instruction to maintain eye-closure and ensure participants were not asleep. This protocol is referred to as split-block recording. All recordings were performed as part of a longer testing session, involving additional EEG paradigms and were counterbalanced within this.

Data was recorded using appropriately sized EGI hydrocel high density sensor nets (containing 128 channel silver–silver chloride electrodes). Electrodes above, below and beside the outer canthus of each eye recorded vertical (VEOG) and horizontal (HEOG) electro-occulogram, respectively. The EEG signal was referenced to the vertex during recording, and signals were recorded using a bandpass filter of 0.1–100 Hz, amplified using a gain of 10 000, and sampled at a rate of 250 Hz. Recordings were made using NetStation (Electrical Geodesics, Inc., Eugene, OR). Electrode impedances were maintained below 50 kΩ. Data is available upon request.

All EEG preprocessing was performed using EEGLAB (Delorme and Makeig 2004) for MATLAB (MathWorks, Natick, MA). The continuous EEG signal was digitally filtered using a low pass filter of 30 Hz. All data obtained from six channels situated around the ears were removed due to poor fit of these channels during recordings as a result of morphological differences in those with DS. As there was a high degree of variability in blink artifacts between participants, and as there is no clear validated algorithm approach to artifact removal in DS, movement and/or blink artifacts were removed manually (i.e., affected data excluded) based on visual inspection. Bad channels were also identified based on visual inspection and were replaced using spherical spline interpolation (SSI; Perrin et al. 1989); a widely used method for estimating missing data values in arrays with more than 65 electrodes (Ferree 2006). It estimates missing data using spatially weighted existing values that are approximated to positions on a sphere (Ferree 2006; Kang et al. 2015). In this study, the mean number of channels interpolated per participant was 1.82 (range 0–5). Remaining channels were re-referenced to the average electrode (with the exception of VEOG and HEOG channels, which were removed from analysis following manual removal of blink artifacts). Datasets were segmented into 2-s epochs. Participants with fewer than 12 such epochs were excluded from further EEG analysis. This threshold was chosen as a pragmatic trade-off between maximizing the availability of artifact free data, while attempting to obtain stable power distributions in the frequency band of interest. The average power spectral densities estimated from this threshold were conserved at the subject level (see Supplementary Figs S1 and S2).

### Spectral Analysis Procedure

Spectral estimates were obtained using multitaper analysis for each channel. Multitaper estimates of spectral power were calculated across 2-s windows using a time resolution of 400 ms with steps of 50 ms and a bandwidth of 3 dB. Estimates were then averaged to within time windows and across time windows for each subject, and average scalp maps were used to estimate the SPM results. Scalp maps were generated using a development version of SPM12 (12.3, updated 03/08/2018) for MATLAB and were spatially smoothed to minimize the effects of spatio-anatomical differences between participants (Gaussian smoothing kernel 2*2px across the 64*64 pixel scalp map). SPMs were thresholded with family-wise error correction at *P = 0.05*.

Mean scalp maps of theta (5 Hz) and alpha (8 Hz) power were generated to assess their regional distribution and determine the location of maximum power in each band. We then used linear regression to examine the relationship between raw KBIT-2 score and both alpha peak amplitude (i.e., the maximum power within the 8–13 Hz range) and APF (i.e., the frequency within the 8–13 Hz range at which peak amplitude occurs) in the spectra derived from regional electrode averages (occipital E70, E71, E74, E75, E76, E82, E83; frontal E4, E5, E10, E11, E12, E16, E18, E19). Finally, a scalp-wide SPM of power in the combined theta–alpha (4–13 Hz) range was generated, and regression was used to identify significant associations between raw KBIT-2 score and theta–alpha power across the scalp (*P* < 0.05, with family-wise error correction). This analysis was conducted using a general linear model (Kiebel et al. 2005).

### Dynamic Causal Modeling Procedure

The results from the above analyses of scalp (sensor space) data motivated a DCM of a distributed bilateral alpha network (in source space). This analysis followed a hierarchical model inversion: (1) for each subject, a network model of coupled neural-masses (i.e., the CMC model (Moran et al. 2013)) was inverted to explain the complex cross-spectra of oscillatory activity across the scalp; that is both power distribution within different frequency bands, and the phase relationship between them; (2) between-subject effects at the level of the subject-specific network connectivity (DCM) parameters were estimated using a parametric empirical Bayesian (PEB) approach (Friston et al. 2016). At this between-subject level, we used KBIT-2 raw score, age, and their interaction as regressors of interest, in addition to two noise regressors (counterbalancing order and whether the paradigm was split block or whole block). DCM for cross-spectral density—as applied here—enables the inference and estimation, within a Bayesian framework, of directed coupling (effective connectivity) among key sources in this network, as well as the parameters (time constants, intrinsic connection strengths) that define local circuitry. This particular form of DCM and has been validated in a range of studies of distributed networks in EEG (Boly et al. 2012; Legon et al. 2016; Symmonds et al. 2018).

Nodes of interest were chosen *a priori* based on imaging literature analyzing EEG activity in combination with functional magnetic resonance imaging (fMRI) and included bilateral occipital, parietal, and frontal sources as a putative alpha network. Specifically, Laufs et al. (2003) examined correlations between BOLD signal and alpha power. Associations were found in the primary visual cortex (V1), superior parietal lobule (SPL), and middle frontal gyrus (MFG). These nodes were selected for the current study. Although further nodes in the alpha network have also been identified (e.g., subcortical structures (Omata et al. 2013)), the influence of these sources are accounted for within the DCM.

Age was orthogonalized to examine variance not explained by KBIT-2 score in order to increase statistical efficiency (a linear model between raw KBIT-2 score and age was fit and the residuals of this linear fit were retained). Age was chosen as the variable to be orthogonalized because general cognitive ability was the main outcome of interest in this study.

To optimize prior expectations for the use of the CMC model generating alpha oscillations, we took a two-step approach: (1) based on the simulated spectra of a single CMC, we optimized several time constants (T1, 2, 3) and decreased population variance (*S* parameter) in order to allow for power spectra that contain a frequency peak in the alpha range. (2) Using those prior parameter values, we then inverted all subjects—effectively scanning the parameter space around the above priors by inverting different subjects. From this pool of inverted subjects, we selected the posterior parameter estimates of a representative subject for whom the inversion provided good model fits. Finally, these values were used as priors for each subject during a second inversion that was the basis for further analysis. The ensuing prior used for the model inversion of each subject are shown in Table 1.

**Table 1:** *T*_1_—superficial pyramidal cell time constant; *T*_2_—inhibitory interneuron time constant; *T*_3_—spiny stellate cell time constant; *T*_4_—deep pyramidal cell time constant; *g*_1_—superficial pyramidal cell modulatory self-connection; *g*_2_—superficial pyramidal cell to spiny stellate cell inhibition; *g*_3_—inhibitory interneuron to spiny stellate cell inhibition. Note that *T* and *s* parameters only have one value that is applied to all regions.

Parameter	Left V1	Right V1	Left SPL	Right SPL	Left MFG	Right MFG
*τ*_1_	2.17	=	=	=	=	=
*τ*_2_	2.94	=	=	=	=	=
*τ*_3_	3.81	=	=	=	=	=
*τ*_4_	0.66	=	=	=	=	=
*g*_1_	−0.39	−0.05	−0.33	0.18	−0.14	−0.03
*g*_2_	−0.33	0.22	0.84	0.73	0.42	−0.08
*g*_3_	−0.05	−0.05	−0.23	−0.54	−0.31	−1.67
*s*	−0.63	=	=	=	=	=

We compared a subset of models with variations in a subset of the parameters explaining the between-subject model regressors. This model comparison can be done efficiently by applying Bayesian Model Reduction estimates (Penny et al. 2004; Friston et al. 2015). Although Bayesian model reduction can be employed to search the model space exhaustively (i.e., compare all possible combinations of model parameters), we chose here to focus on a selection of models that were informed by our neurobiological questions. These models focused on the type of connectivity (i.e., intrinsic, forward, and backward connections), as these connections are mediated by distinct neurotransmitters (AMPA, NMDAR, and GABA transmission, respectively, for Forward, Backward, and Intrinsic connections) in our model. For inference on this more restricted model space, we are harnessing Bayesian model reduction for its computational efficiency.

Overall candidate models differed in terms of whether forward, backward, and intrinsic self-inhibitory connections showed cognitive and age-related effects. The model with the greatest log-evidence (as approximated by negative free-energy) was considered the winning model. Connectivity changes associated with KBIT-2 score in the winning model were then examined to quantify the microcircuit correlates of cognitive ability and thereby explain the observed differences in theta–alpha power in terms of neuronal excitation–inhibition balance.

**Figure 1.**
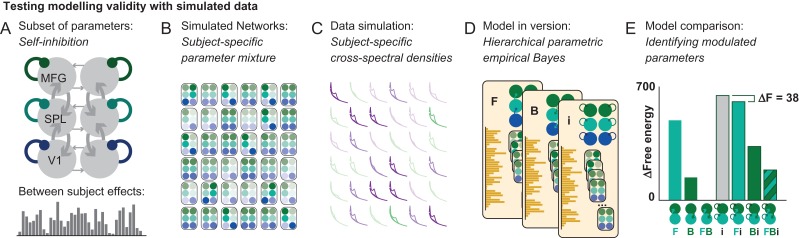
Assessing construct validity of the parametric empirical Bayesian (PEB) approach. (*A*) A baseline model parameterization is combined with a between-subject random effects regressor by allowing modulation of specific subsets of parameter (here, intrinsic self-inhibitory connections) by the weight given in the between-subject effect regressor. (*B*) This produces 36 different DCM models, that differ in their intrinsic connectivity in a way that is affected by the between-subject regressor. (*C*) These models are used to generate synthetic cross-spectral densities (of which the first three eigenmodes are shown here) that vary systematically with the regressor. (*D*) These data are used as input for DCM model inversion. This approach compares subsets of models where between-subject effects are expressed only in a subset of parameters. (*E*) Using the free-energy measure of model likelihood for each of these models, we can then compare how good an explanation these models offer for the data, and successfully identify the model with changes in intrinsic self-inhibition as the winning model.

We tested the construct validity of using this approach by simulating data from *in silico* six-node networks, where a between-subject regressor modulated specific model parameters (Fig. 1). We generated a baseline model, and a random between-subject regressor. Using these, we then generated 36 single-subject models, where intrinsic inhibitory connections were modulated according to the weight given by the between-subject regressor and simulated output cross-spectral densities that would be observed from the particular model specifications. Using hierarchical PEB inversion of dynamic causal models described above, we then inverted models based on these data, and assessed the evidence that the data were generated from different “reduced” models—that is, models that only allow between-subject variation to be caused by specific subsets of model parameters. In this (group-level) model comparison, we can successfully identify the known generative parameter variations. These simulation results further support the use of computationally efficient estimation of log-model evidence using Bayesian model reduction, which has been successfully employed across multiple complex network analyses in EEG (Friston et al. 2016; Rosch et al. 2018; Symmonds et al. 2018).

## Results

### Spectral Analysis Results

**Figure 2.**
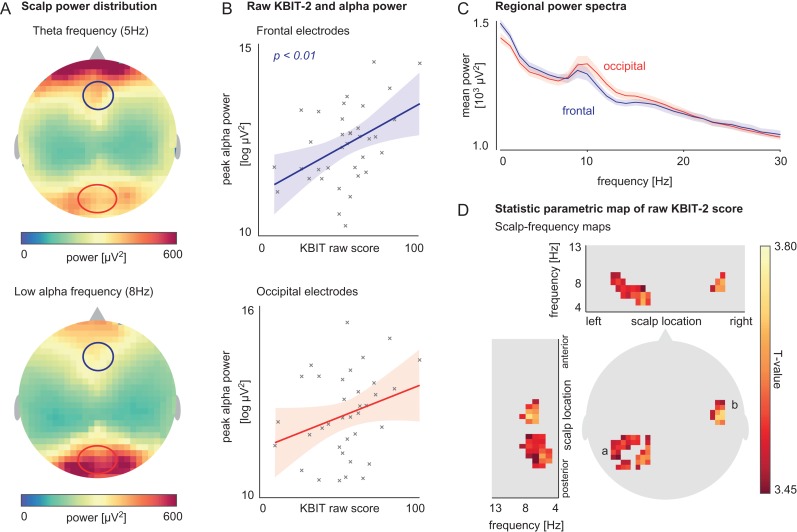
*A*. Mean scalp map of power distribution for theta (5 Hz, top) and low-alpha (8 Hz, bottom) activity. The frequencies shown here are exemplary and chosen for their complementary distribution across the scalp (all statistical inference is made across the whole theta–alpha spectrum). *B*. Linear relationship between peak alpha amplitude at frontal and occipital regions (eight channel average frontal; seven channel average occipital) and raw KBIT-2 score. *C*. Mean regional power spectra at frontal and occipital regions (regional channel average). *D*. T-statistic scalp map showing voxels with a significant relationship between raw KBIT-2 score and power in theta–alpha (4–13 Hz) range; voxels were located over left occipital (*a*) and right temporal (*b*) scalp regions.

Mean raw KBIT-2 score was 54.84 (19.64 SD; 10–102 range). Average scalp maps and mean power spectra by region for all participants (*n* = 36) showed frontal dominance of theta activity as well as the expected posterior alpha distribution (Fig. 2*A*,*C*). Linear regression revealed peak alpha **amplitude** at the frontal region to be significantly associated with raw KBIT-2 score (*t*(34) = 2.93, *P* = 0.006; Fig. 2*B*). The relationship between peak alpha **amplitude** at the occipital region and raw KBIT-2 score did not reach significance (*t*(34) = 1.77, *P* = 0.085; Fig. 2*B*). In our sample, no significant association between peak alpha **frequency** and KBIT-2 was found for frontal (*t*(34) = 0.04, *P* = 0.96), or occipital electrodes (*t*(34) = 1.03, *P* = 0.31) (results not shown).

Whole-scalp SPM analysis of EC spectral estimates revealed clusters of significant positive correlations between estimated general cognitive ability (raw KBIT-2 score) and power in the 4–13 Hz range. Clusters were located over left occipital and right temporal scalp regions (Fig. 2*D*).

### Dynamic Causal Modeling Results

**Figure 3.**
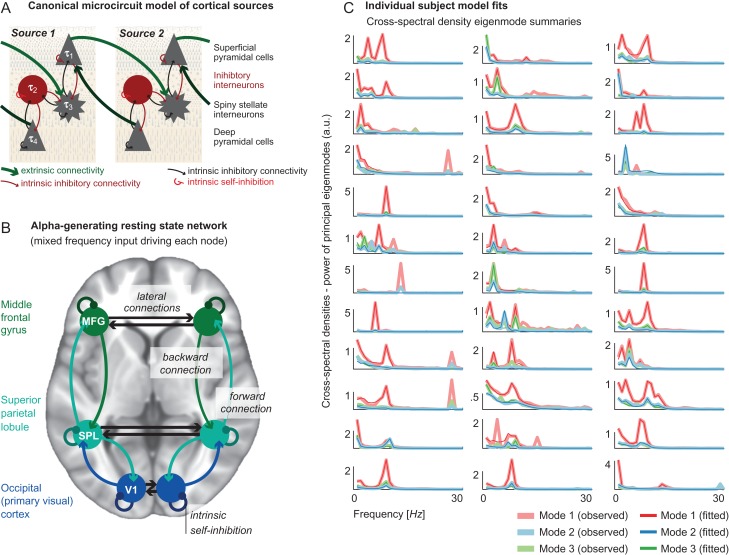
*A*. Schematic illustrating the canonical microcircuit (CMC) neural mass model, which comprises four distinct neural-masses mapped onto specific cortical layers. The model includes between-population (local, intrinsic) excitatory (black) and inhibitory (red) connections and within-population intrinsic self-inhibition; as well as between-source (long-range, extrinsic) excitatory connections. *B*. Diagram showing the bilateral network model, including nodes within the primary visual cortex (V1), superior parietal lobule (SPL), and middle frontal gyrus (MFG), also indicating extrinsic forward, backward, and lateral connections; as well as intrinsic self-inhibition. *C*. Individual subject model fits showing the top three eigenmodes decomposed from whole-scalp data alongside the model fits for each of the 36 participants.

We identified cortical sources that are associated with increased alpha power from the literature. Specifically, we used Montreal Neurological Institute (MNI) coordinates of fMRI clusters reportedly associated with EEG alpha power derived from EEG/fMRI experiments (Laufs et al. 2003) as priors for the DCM analysis (Fig. 3*B*). Based on this literature, we identified three bilateral sources: primary visual cortex (V1, MNI coordinates [*x*,*y*,*z*] left: [−16,−92,0]; right: [12,−92,21]), SPL (MNI coordinates left: [−48,−56,52]; right: [34,−51,39]), and MFG (MNI coordinates left: [−46,37,16]; right: [46,18,21]). Coordinates that were reported as Talairach coordinates in the original report were converted to MNI coordinates using an online conversion tool (http://sprout022.sprout.yale.edu/mni2tal/mni2tal.html, accessed 03/07/2018), which itself is based on a previously published conversion (Lacadie et al. 2008).

Each node was modeled as an EEG dipolar source, with activity being generated by a CMC neural mass model (Fig. 3*A*). This model was fitted to complex cross-spectral density summaries of ongoing EEG oscillations that preserved both amplitude and phase relationships. The priors of the CMC were tuned to generate alpha oscillations and were identical for each participant. The model fits for individual DCMs inverted to subject-specific spectra are shown in Figure 3*C*; specifically, the DCM principal eigenmode summary of the whole-scalp data from each participant (here, with eight eigenmodes—this is standard practice in DCM and provides a well-tested balance between the richness of the dataset (preserving components for analysis) and allowing model inversion (reducing the dimensionality of multi-channel recordings)). This figure shows the top three eigenmodes of the empirical recordings versus those generated by the DCMs (Fig. 3*C*), illustrating an almost universally excellent fit in the <15 Hz spectra (faster frequencies have not been captured as accurately because the model was optimized to generate <15 Hz activity).

**Figure 4.**
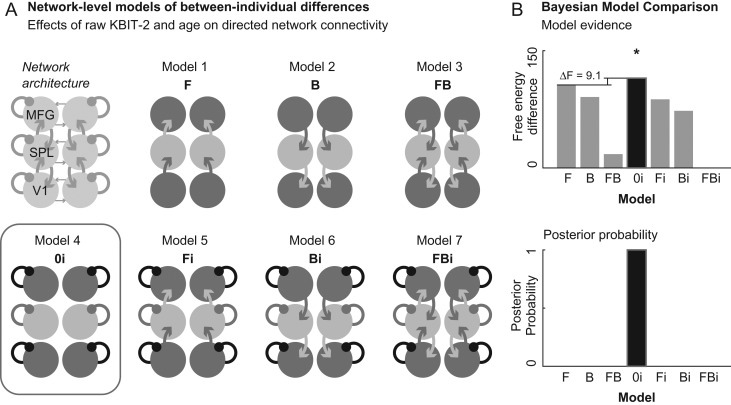
*A*. Diagram showing the seven network-level candidate models, which differed in terms of which forward (F), backward (B), and intrinsic self-inhibitory (i) connections were affected by between-subject differences in cognitive ability and age. Red box denotes the (intrinsic self-inhibition) model with the greatest model evidence *B*. (Log) model evidence (negative free-energy) for each of the seven candidate models. Model 0i (model 4; intrinsic self-inhibition alone) shows the highest negative free-energy, with a negative free-energy difference to the next highest model of 9.1 (top diagram) and a posterior probability of ~1 (bottom diagram).

We then compared seven candidate models (Fig. 4*A*), where between-subject effects were only allowed to affect a subset of possible model parameters (combinations of extrinsic and intrinsic connectivity parameters). Bayesian model selection identified the model allowing for intrinsic self-inhibition alone as having the greatest model evidence. This model had a negative free-energy difference to the next highest model of 9.1 (corresponding to a Bayes Factor of over 100) (Fig. 4*B*), which is considered very strong evidence for the model compared to its alternatives (Kass and Raftery 1995).

**Figure 5.**
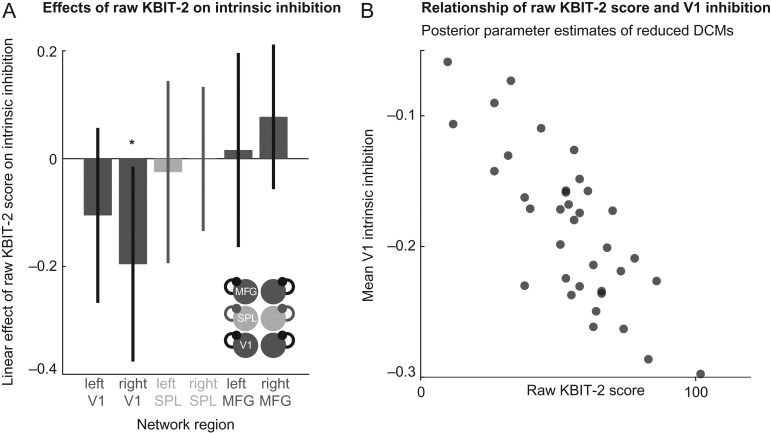
*A*. Bar chart showing the linear (between-subject) effect of raw KBIT-2 score on intrinsic self-inhibition for each node in the network of the winning model (allowing for changes in intrinsic self-inhibition alone; shown bottom right). Bayesian confidence intervals indicate that the effects estimated with most certainty are located at right V1. *B*. Scatter graph showing inverse correlation between raw KBIT-2 score and mean V1 (left V1 and right V1 average) intrinsic inhibition of the winning model (i.e., the model with the highest model evidence in model space).

The variation of connectivity parameters best explained by KBIT-2 scores in the winning model architecture (i.e., restricting between-subject effects to intrinsic self-inhibition) is shown in Figure 5*A*. The biggest effect (which was also estimated with the greatest certainty) was seen in right V1, where a higher raw KBIT-2 score was associated with less intrinsic-inhibition (i.e., disinhibition). This negative linear relationship between (mean bilateral) V1 self-inhibition and KBIT-2 raw score is further illustrated in Figure 5*B*, showing mean V1 intrinsic inhibition versus raw KBIT-2 score plotted for each subject. Note the Bayesian confidence intervals delimit the certainty with which each parameter is estimated. This shows that individually some parameters are more identifiable during the model inversion (i.e., their exact value can be estimated more precisely than others). However, the statistical test of our hypotheses was performed during the Bayesian model selection (i.e., by comparing models where different sets of parameters were included to explain the variation between subjects, we identified an optimum model that balances complexity and model accuracy). This means that there is strong statistical evidence for the inclusion of all the parameters shown, even where the individual parameter values cannot be estimated with high certainty (as indicated by larger Bayesian confidence intervals).

There were no effects of age or the interaction between KBIT-2 raw score and age that survived Bayesian Model Reduction.

## Discussion

We show that general cognitive ability in adults with Down syndrome (DS) is associated with particular, distributed signatures in scalp activity across theta–alpha frequency ranges. Furthermore, DCM indicates that across our model space, between-subject differences in intrinsic self-inhibition producing the differing EEG spectra are associated with individual differences in general cognitive ability. In particular, we demonstrated that higher cognitive ability was associated with higher alpha peak amplitude at frontal electrodes and higher theta–alpha band power in distributed regions across the scalp. Within a generative model of a distributed alpha generating network, these changes were associated with lower intrinsic self-inhibition in bilateral V1 and an implicit loss of E/I balance maintained by inhibitory interneurons.

The importance of alpha activity in cognition is highlighted by work in the TD population relating alpha oscillations to attention and memory processes (Klimesch 1999; Richard Clark et al. 2004; Palva and Palva 2007; Samaha and Postle 2015; Foster et al. 2016; Oswald et al. 2017; Voytek et al. 2017). Within the DS population, a limited number of studies have reported associations between alpha power and cognitive ability. Similar to findings of the present study, Medaglini et al. (1997; 45 adults with DS) reported a significant positive correlation between alpha power (8–12 Hz) and cognitive ability (attention and memory test performance, as measured by the Cancellation Task and the Rivermead Behavioural Memory Task, respectively). In contrast, a smaller study by Politoff et al. (1996; 13 adults with DS) reported a significant negative correlation between low-alpha power (8.8 Hz) and cognitive ability (as measured by the Picture Absurdities Test). This study, however, was restricted to higher functioning participants and may therefore not be comparable to the present study. Interestingly Velikova et al. (2011; 25 individuals with DS) reported cognitive ability (as measured by the Wechsler Adult Intelligence Scale and the Rivermead Behavioural Memory Task) was positively associated with power at the high-alpha range (11–12 Hz) and negatively associated with power at the low-alpha range (7–8 Hz). Individuals with cognitive decline were not excluded from this study, however, which may be a potential source of inconsistency between the low-alpha findings reported here. A particular strength of our study is the exclusion of adults with noticeable cognitive decline, which allows pre-decline relationships between oscillatory correlates and cognitive ability to be determined. Cognitive decline and dementia represent disease processes that warrant future specific investigation.

The association between higher general cognitive ability and lower intrinsic self-inhibition demonstrated here is in keeping with animal model literature demonstrating markers of over-inhibition in the Ts65Dn mouse (a commonly used mouse model of DS) compared to wildtype mice (see Contestabile et al. (2017) for review). This has included an increased number of GABAergic interneurons, enhancement of interneuron excitability, and reduced glutamatergic transmission (Chakrabarti et al. 2007; Hernández et al. 2012; Tyler and Haydar 2013; Hernández-González et al. 2015; Contestabile et al. 2017). Treatment of Ts65Dn mouse models with pharmacological agents that reduce inhibition has also been shown to improve memory deficits in these animals (Braudeau et al. 2011). Based on findings from the mouse model literature, over-inhibition has also been the target of recent human drug trials aimed at improving cognition in individuals with DS. Thus, far these trials have either been unsuccessful (Roche; ClinicalTrials.gov Identifier: NCT02024789) or are ongoing (Balance Therapeutics). The unsuccessful study by Roche was a phase II placebo-controlled trial of an inverse agonist of α5 subunit-containing GABAA receptors (basmisanil). No improvement in cognitive function was observed over 26 weeks in participants (aged 12–30). Importantly, different inhibitory pathways across the brain express different GABAA receptor subunits—α5 containing receptors are highly expressed in the adult hippocampus but have low expression in other brain areas (Vargas-Caballero et al. 2010). As atypicalities in the brains of individuals with DS are not localized to the hippocampus, alternative targets of over-inhibition require investigation. The findings of our study—indicating a relationship between localized cortical over-inhibition and cognitive ability—further support this.

It is of further interest that human studies using postmortem tissue, magnetic resonance spectroscopy (MRS), and neural progenitor cells have failed to find evidence of over-inhibition in DS and have instead indicated under-inhibition may be present. The indication in frontal regions of a positive relationship between inhibition and cognitive ability in this study, as opposed to the negative relationship in V1, is suggestive of potential regional differences in E/I. It is possible such differences contribute to inconsistencies in the literature and suggest the over-inhibition narrative in DS may in fact be more complex, with regional differences also being important to consider. It follows that MRS studies may prove useful for examining E/I mechanisms in DS further, with studies focusing on different brain regions. The nodes identified in this study may inform targets for this.

Mechanistically, the parameter of intrinsic inhibition identified here describes recurrent self-connections that dampen the excitability of the large projection neurons in the circuitry of the CMC (Fig. 3*A*). This is a population level summary of intra-laminar local inhibitory populations that connect pyramidal cells within the supragranular, or infragranular cortical layers. Less intrinsic inhibition, as seen in V1 with higher KBIT-2 scores, therefore releases the self-suppression of ongoing activity, and results in more excitable cortical sources. It could be hypothesized that reduced inhibition at a cellular level would lead to increased electrophysiological activity in this region; manifesting as release of synchronous alpha activity across the network as measured by EEG (a desirable outcome during eye-closure and indicative of efficient network-level control). Conversely, increased V1 intrinsic inhibition (as seen in individuals with lower KBIT-2 scores) may therefore attenuate the alpha that can be measured in the EEG across the whole scalp.

It is noteworthy that adults with DS show reduced alpha power when compared to age-matched TD controls (Ono et al. 1992; Murata et al. 1994; Locatelli et al. 1996; Babiloni et al. 2009). The relationship between alpha and general cognitive ability in adults with DS reported here therefore indicates that within individuals with DS, those with an EEG spectrum closer to that of individuals from the TD population (i.e., higher alpha peak amplitude) are also closer to individuals from the TD population in terms of general cognitive ability.

This is the first study to examine parameters of E/I in humans with DS. Our results suggest regionally specific modulation of intrinsic self-inhibition as a potential therapeutic target for cognitive enhancement in DS. Recent research has demonstrated the utility of transcranial direct current stimulation in modulating local E/I balance in order to enhance memory through reducing local GABA levels in the TD population (Barron et al. 2016). The localized nature of these findings, although problematic for pharmacological manipulation, lends itself to such targeted approaches. A further potential practical implication of this study is the use of non-invasively measured V1 intrinsic-inhibition as a biomarker of general cognitive ability, which drug trials in DS may find useful.

It is important to note, however, that the differences in intrinsic inhibition between adults with DS reported here may be compensatory responses to a backdrop of altered neurobiology, rather than a direct consequence of trisomy 21. For some individuals, it may be the case that excess inhibition provides an advantage of some form (for example, reducing seizure-like activity, which is more common in DS (Pueschel et al. 1991)). Caution should therefore be taken when considering intrinsic inhibition as a potential therapeutic target in this population.

In the current study, age was not identified as an important factor associated with theta–alpha activity. Despite this, age-related changes in alpha activity are commonly reported in DS literature (e.g., Johanson et al. 1991; Soininen et al. 1993; Katada et al. 2000). It is possible that age was not an important factor in the current study because of the relatively young mean age of adult participants (30.92 years), before substantial AD-associated pathology in adults with DS is likely to occur (AD pathology is present in the brains of almost all adults with DS over the age of 30 (Wisniewski et al. 1985; Mann 1988)). Orthogonalizing age with respect to KBIT-2 score may have also reduced a possible relationship. Alternatively, it is possible that when cognitive decline is controlled for (as in the present study), age is not an important factor associated with theta–alpha activity.

Other studies using neural mass computational models to investigate resting-state alpha activity have highlighted the importance of inhibitory interneurons within the Lateral Geniculate Nucleus of the thalamus (Bhattacharya et al. 2016). Research suggests these neurons play a role in maintaining homeostatic balance within the network by suppressing any instability that may arise from anomalous synaptic activity (e.g., dysrhythmia or slowing) (Bhattacharya et al. 2016). It is therefore possible that the parameter of intrinsic self-inhibition in this study may play a similar role in maintaining homeostatic balance within the alpha network. It could be argued that maintaining excitation–inhibition balance within this network may be a greater challenge for the DS brain, where numerous sources (from biochemical to a gross anatomical) may contribute to anomalous syntactic activity.

In order to achieve good model fits, we adjusted CMC parameters so that individual CMC sources would provide a frequency peak in the alpha range. This approach means that we may have already “explained away” some of the whole-scalp observations through adjustment of local parameters, meaning that long-range connectivity may be less likely to emerge as a true experimental effect in the results. However, the DCM for cross-spectral densities largely relies on the complex component of the cross-spectra and thus the phase relationship between sources to infer underlying long-range effective connectivity between sources. As this was not included in the optimization of the priors, we would not have biased against the main data features that allows inference on long-range connections.

It remains unclear why alpha peak amplitude was significantly associated with KBIT-2 score in frontal regions, yet for DCM analysis both the effect size of—and the confidence in—the association of KBIT-2 score and intrinsic inhibition were highest in the occipital regions. Because there is a non-linear mapping between model parameters and EEG spectral features, whilst non-intuitive, this does nevertheless reflect the evidence in the data. The dynamics of the network of sources—as modeled here—depends not only on the dynamics of isolated microcircuits, but also of the integrated and distributed dynamics of all coupled sources. Thus, a source level change in intrinsic connectivity in the occipital nodes may have little effects on the observable node dynamics (because of the nonlinear mapping); yet, a slight change in the input from occipital sources to frontal sources may yield larger observable effects in sensor space.

This highlights the fact that it is difficult to interpret sensor space (scalp) effects in terms of the underlying causes in source (brain) space. It is possible that our findings were influenced by the large extent of inter-individual variability in EEG measures and network parameters reported here; however, the PEB approach used here specifically accommodates random between-subject effects. Larger studies may therefore help interpret this further. Future studies would also benefit from using participant MRIs to localize nodes at an individual level.

A general aspect of Bayesian model inversion is the reliance on a set of prior assumptions (i.e., the activity generated by the model arises from CMC optimized to generate activity of interest within predefined nodes of interest). Despite optimizing the model with Bayesian model reduction, other factors not accounted for by the structure of the models entertained may also be important—and potentially result in models with greater evidence.

Oscillatory correlates of individual differences in cognitive ability in other forms of ID do not appear to have been studied; however, atypical connectivity in theta and alpha bands have been demonstrated in adults with fragile X syndrome (van der Molen et al. 2014). It therefore remains to be seen whether the findings reported here are unique to individuals with DS or are related to ID in general. Future research with non-DS ID populations are necessary to clarify this.

Future studies would benefit from recruiting older individuals in order for relationships between ageing and model parameters to be fully examined. During this study, we found practical difficulties related to traveling to our testing location were common in older individuals with DS. The use of portable EEG equipment may therefore increase participation of older adults. Furthermore, larger or targeted studies will enable associations between Apolipoprotein E *(APOE)* genotype and EEG activity to be explored, with associations with *APOE* genotype being of particular interest due to the known increased risk of AD in those possessing the *APOE* ε4 allele. As these findings are based on a single EEG paradigm, modeling EEG activity using DCM for other paradigms (e.g., the auditory oddball) would help improve the validity of conclusions. Finally, DCM can also be used to model the effects of specific pharmacological compounds on the network and parameters identified here. Such an approach may aid in drug discovery.

## Supplementary Material

Supplementary Data

## Appendix

**Glossary**

**Adult**: used in this study to refer to individuals aged 16 and over (i.e., not a child).

**Age-adjusted IQ score**: when a raw score on a general cognitive ability test is converted to a standardized score (using population norms) according to participant age to provide an estimate of intelligence quotient (IQ).

**Bayesian model selection (BMS)**: a method for determining the most likely hypothesis (among a set of competing hypotheses) about the mechanisms that generated observed data.

**Dynamic causal modeling (DCM)**: a framework for fitting differential equation models of neuronal activity to brain imaging data using Bayesian methods.

**Eigenmode:** a frequency component of a signal. Principal eigenmodes represent the main components of the signal and can be used to summarize data.

**Free-energy**: the function that is optimized during model inversion in DCM. Log-evidence (as approximated by negative free-energy) is used to determine the winning model.

**KBIT-2 score**: Kaufmann’s Brief Intelligence Test second Edition (KBIT-2) raw test score (Kaufman and Kaufman 2004). Used to provide an estimate of general cognitive ability.

**Model inversion**: method for fitting the data to the model to estimate model parameters. The method involves minimizing free-energy/maximizing negative free-energy in order to maximize the model evidence or marginal likelihood.

**Posterior probability**: within Bayesian statistics, posterior probability is the statistical probability that a hypothesis is true, given the relevant evidence.

**Typically developing (TD)**: used in this study to refer to individuals who do not have a neurodevelopmental disorder.
