# Supplementary material for: Dynamic Causal Modeling of the Relationship between Cognition and Theta–alpha Oscillations in Adults with Down Syndrome
Source: Cereb Cortex. 2019 Mar 16;29(5):2279–90. doi: 10.1093/cercor/bhz043 (PMC6458903; doi:10.1093/cercor/bhz043)
Supplement: Supplementary Data [file bhz043_supplementary_materials.zip › bhz043_SUPPLEMENTARY.docx]

|  |
| --- |
| ***Supplementary Figure S1:*** *This figure shows Pearson’s correlation coefficients between theta-alpha power spectral densities estimated from repeated 2 second window samples from a single participant. Samples contained increasingly large numbers of 2 second segments, ranging from 1 (left) to 24 (right). At the chosen cut off of a minimum of 12 segments, variability decreases, and between-sample correlations stabilize at > 0.9.* |

***Supplementary Figure S2:*** *Each plot shows the correlation of power spectral densities estimated from half the available trials against the other half. This shows high internal consistency of the available samples.*
